# Supplementary material for: From sequence to enzyme mechanism using multi-label machine learning
Source: BMC Bioinformatics. 2014 May 19;15:150. doi: 10.1186/1471-2105-15-150 (PMC4229970; doi:10.1186/1471-2105-15-150)
Supplement: Additional file 2 — Java code of ml2db. Additional file ml2db_code.tar.gz contains the Java source code to run the multi-label machine learning experiments and save the results to database. The code’s Javadoc is included. [file 1471-2105-15-150-S2.zip › additional file 2/ml2db/ml2db/doc/main/ml2db/al/utility/pickers/byfrequency/AttSetByFrequencyPicker.html]

AttSetByFrequencyPicker


JavaScript is disabled on your browser.


- Overview
- Package
- Class
- Use
- Tree
- Deprecated
- Index
- Help

- Prev Class
- Next Class

- Frames
- No Frames

- All Classes

- Summary:
- Nested |
- Field |
- Constr |
- Method

- Detail:
- Field |
- Constr |
- Method


main.ml2db.al.utility.pickers.byfrequency

## Class AttSetByFrequencyPicker

- java.lang.Object
- - main.ml2db.al.utility.UtilityMetric
  - - main.ml2db.al.utility.pickers.RandomPicker
    - - main.ml2db.al.utility.pickers.byfrequency.ByFrequencyPicker
      - - main.ml2db.al.utility.pickers.byfrequency.AttSetByFrequencyPicker

- All Implemented Interfaces:
  :   Picker

  ---

    

  ```
  public class AttSetByFrequencyPicker
  extends ByFrequencyPicker
  ```

  Serves the instances in order of frequency of their attribute sets (most
  frequent first). After serving one instance for each attribute set, it starts
  over again, again starting with the most frequent.

  Version:
  :   29 Apr 2012

  Author:
  :   Luna De Ferrari ldf(at)st-andrews.ac.uk or
      luna.deferrari@st-andrews.ac.uk

- - ### Field Summary

    - ### Fields inherited from class main.ml2db.al.utility.pickers.RandomPicker

      `m_debug, m_test`
  - ### Constructor Summary

    Constructors

    Constructor and Description

    `AttSetByFrequencyPicker()`
  - ### Method Summary

    Methods

    | Modifier and Type | Method and Description |
    | --- | --- |
    | `java.lang.String` | `getMetricName()` |

    - ### Methods inherited from class main.ml2db.al.utility.pickers.byfrequency.ByFrequencyPicker

      `calculateFrequency, getFeaturesByDescendentFrequency, getFrequencyToFeatureMap, initialise, orderAttsetByDescFreq, setFeaturesByDescendentFrequency, setFrequencyToFeatureMap`
    - ### Methods inherited from class main.ml2db.al.utility.pickers.RandomPicker

      `getInstanceToFeaturesMap, getQueries, getQuery, getStep, getUtility, isDebug, isTest, refresh, setDebug, setFeatureToInstanceMap, setStep, setTest`
    - ### Methods inherited from class main.ml2db.al.utility.UtilityMetric

      `getUnlabelled, setUnlabelled`
    - ### Methods inherited from class java.lang.Object

      `equals, getClass, hashCode, notify, notifyAll, toString, wait, wait, wait`

- - ### Constructor Detail


    - #### AttSetByFrequencyPicker

      ```
      public AttSetByFrequencyPicker()
      ```
  - ### Method Detail


    - #### getMetricName

      ```
      public java.lang.String getMetricName()
      ```

      **Specified by:**
      :   `getMetricName` in class `ByFrequencyPicker`


- Overview
- Package
- Class
- Use
- Tree
- Deprecated
- Index
- Help

- Prev Class
- Next Class

- Frames
- No Frames

- All Classes

- Summary:
- Nested |
- Field |
- Constr |
- Method

- Detail:
- Field |
- Constr |
- Method
